# Antifungal peptides: From modes of action to synergistic and immunologic potential

**DOI:** 10.15698/cst2026.01.315

**Published:** 2026-01-30

**Authors:** Didac Carmona-Gutierrez, Maria A. Bauer, Katharina Kainz, Martin N. Odabas, Frank Madeo

**Affiliations:** 1 Institute for Molecular Biosciences, NAWI Graz, University of Graz, Graz, Austria.; 2 BioHealth Graz, Graz, Austria.; 3 BioTechMed Graz, Graz, Austria.

**Keywords:** *Candida*, resistance, yeast, antimycotics, drug, azoles, immune system

## Abstract

Fungal infections pose a significant global health threat with rising morbidity and mortality rates. However, the repertoire of effective antifungal drugs remains narrow, a challenge that is further exacerbated by the increasing emergence of (multi)drug-resistant strains. This underscores the urgent need for novel therapeutic strategies. Among them, antifungal peptides (AFPs) have emerged as a promising alternative. AFPs are small, naturally occurring peptides produced by a wide range of organisms, including plants, animals, fungi, and bacteria, as part of their innate immune defense. In addition, synthetic and semisynthetic variants have also been engineered. We here underscore the potential of AFPs as viable candidates for the development of next-generation antifungal therapies. In particular, we advocate their multimodal advantage that spans beyond standalone activity, including their synergistic and immune-regulatory potential.

## INTRODUCTION

Fungal infections (FIs) are increasingly becoming a global health burden with devastating socioeconomic consequences 1. In addition to increasing healthcare costs, invasive human mycoses result in high morbidity and mortality rates, resulting in at least 3.8 million deaths worldwide every year 2. Many more millions are affected by critical FIs with severe morbidity. Importantly, the number of cases continues to constantly rise, and the incidence and prevalence of FIs are boosting to alarming levels 2. This development is largely driven by a growing at-risk population, which is mainly comprised of immunocompromised individuals, such as those with HIV/AIDS, cancer, organ transplants, diabetes, and chronic respiratory diseases, but also of the elderly population, which has strongly increased thanks to medical and social advances. At the same time, the application of immune-suppressing drugs and excessive antibiotic use create favorable conditions for FIs 3, 4. Additionally, medical devices like catheters and cardiac valves can not only serve as portals of entry into the human body, but also provide surfaces that promote biofilm formation, enhance fungal resistance and thus contribute to high mortality rates 5.

Although a number of pharmacological options for antifungal treatment do exist, they are currently limited to only four distinct chemical classes: azoles, echinocandins, polyenes and pyrimidine analogs 4. However, their feasibility has been constantly decreasing due to the growth of antifungal resistance in previously sensitive strains 4, 6, among other reasons due to their generous prescription as prophylaxis and the agricultural use of medically related antifungal drugs, resulting in cross-resistance. In addition, pathogenic fungi with intrinsic multiresistance, most prominently the species *Candidozymaauris*(formerly referred to as*Candida auris*) 7, 8, or reduced intrinsic susceptibilities like many pathogens commonly known as non-albicans *Candida* (NAC) species 9, are rapidly emerging. Altogether, this has boosted the environmental reservoirs for drug-resistant fungal pathogens. In reaction to these alarming developments, the World Health Organization (WHO) published a list of priority fungal pathogens at the end of 2022 10, emphasizing the critical need for the advancement of new antifungal strategies.

Among these novel therapeutic approaches is the use of antifungal peptides (AFPs). Like other antimicrobial peptides, AFPs are typically short ( < 100 amino acids) and strongly differ in their amino acid sequences. Despite this diversity, they usually carry a net positive charge and hydrophobic regions, which facilitates membrane interactions. The molecular mechanisms operating their antifungal activity involve multiple targets, which reduces the chances of resistance development. AFPs are naturally produced by various organisms - including microorganisms 11–15, plants and animals – to manage interactions with fungi. AFP variants that are either semisynthetic (*i.e.*, based on natural peptides that have been partly modified or completed through chemical synthesis) or fully synthetic (*i.e.*, generated entirely through chemical synthesis without any biological origin) also exist. They are typically designed to enhance pharmacological properties, minimize side effects, and/or reduce the immunogenicity of natural peptides. The Antimicrobial Peptide Database (APD3) 16 currently lists over 1,500 peptides classified as antifungal.

## ANTIFUNGAL PEPTIDES

**Figure 1:**
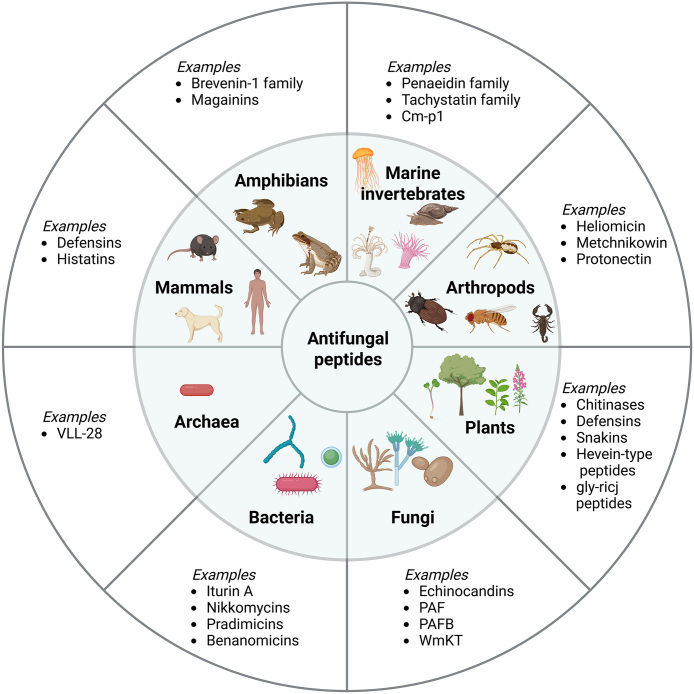
Antifungal peptides are produced across diverse domains of life. Distribution of antifungal peptides (AFPs) among different classes of organisms. Examples of AFPs from each group are provided as a non-exhaustive representation. The classification does not follow strict taxonomic hierarchy and is intended to highlight the broad diversity of AFP sources rather than a comprehensive or systematic categorization. Figure created in BioRender. Odabas, M. (2026) https://BioRender.com/6tuxk9g.

Natural sources of AFPs include microorganisms, plants and animals (Figure 1). Microorganisms, including bacteria, archaea and fungi, release AFPs into the extracellular environment for competitive gain in ecological niches with many of them eliciting activities against a broad spectrum of fungi. The cyclic lipopeptide Iturin A, for example, is produced by *Bacillus subtilis* and has long been known for its potential against multiple fungal pathogens, including *Aspergillus* spp., *Fusarium* spp., and *Penicillium* spp 17, 18. Of note, recent studies have uncovered its possible anticancer effects and hemolytic activity 19, underlining the medical potential of AFPs beyond their antifungal characteristics. Similarly, the first archaeal AFP discovered, VLL-28, also displays antineoplastic potential 20. The antifungal activity of VLL-28, isolated from *Sulfolobus islandicus*, has been shown against numerous clinical isolates of *Candida* spp., both for biofilms and planktonic cells 21. Fungi also produce AFPs themselves. Echinocandins, for instance, like echinocandin B or pneumocandin A0 and B0, are naturally synthesized by different filamentous fungi and have served as basis for the development of clinically relevant semisythetic variants like caspofungin, micafungin, anidulafungin and rezafungin 22, 23. Many other examples for fungal AFPs exist, *e.g.*, PAF and PAFB, which are produced by the ascomycete *Penicillium chrysogenum* and inhibit growth of diverse human pathogenic filamentous fungi and yeasts 24–26.

Multicellular organisms produce AFPs as a first-line defense against fungal attacks. In plants, diverse peptide families like the chitinases, the defensins, the snakins, the hevein-type peptides or the gly-rich peptides have been shown to exhibit wide antifungal activities, mainly against phytopathogenic fungi 27. However, the antifungal properties of some plant AFPs have also been shown to target human pathogenic fungi, underlying their potential for medical applications 28. Animals also produce AFPs as part of their innate immune response to fungal pathogens. Especially in invertebrates, which lack adaptive immunity, AFPs play a crucial role. AFPs from marine invertebrates, for example, include members of the penaeidin and tachystatin families 27 as well as Cm-p1, a short peptide isolated from marine snails, which exhibits antifungal effects against several human fungal pathogens, including *Candida albicans* and *Cryptococcus neoformans* 29. AFPs from insects have also been shown to have antifungal properties against human pathogenic fungi 27, 30, 31. Some examples include protonectin, isolated from the venom of the neotropical social wasp *Agelaia pallipes pallipes* 32, heliomicin from the tobacco budworm *Heliothis virescens* 33, or metchnikowin from the fruit fly *Drosophila melanogaster* 34, 35. In general, arthropods are a rich source of AFPs and can be found, beyond insects, in other members of this phylum, including centipedes 36 or arachnids like spiders and scorpions 37.

Vertebrates rely on AFPs as well. Amphibian skin secretions, for instance, contain numerous antimicrobial peptides, including AFPs 38. Notably, AFPs isolated from toads and frogs have been shown to also be active against *Candida* species, such as the magainins from the African clawed frog *Xenopus laevis* 39 or the brevinin-1 family members from the foothill yellow legged frog *Rana boylii,* 40 among others. Finally, mammalian AFPs encompass several peptide groups, including salivary histatins 41 and mammalian defensins 42–44. The latter ones are further classified into the α - and β -defensin subfamilies based on the arrangement of highly conserved cysteine residues. While α -defensins, also termed human neutrophil peptides (HNPs), are mostly produced by neutrophils, β -defensins are particularly abundant in epithelial cells across multiple organs 14.

## MODES OF ACTION OF AFPs

**Figure 2:**
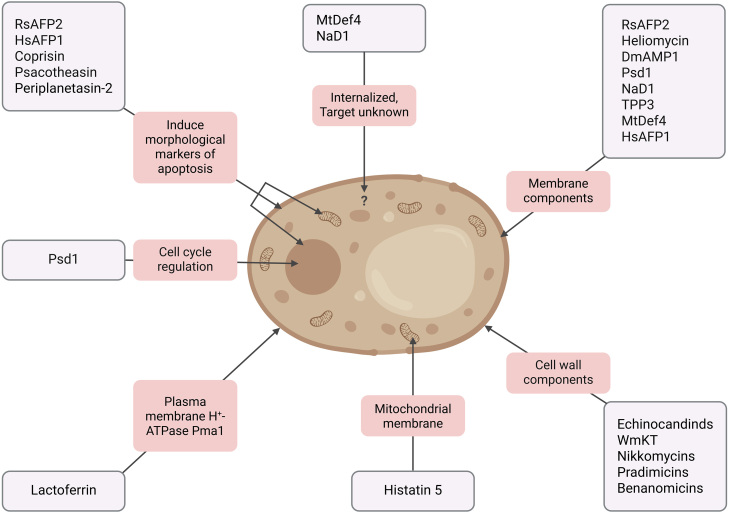
Mechanistic routes of antifungal peptides. Cellular structures and processes targeted by antifungal peptides (AFPs). Examples of AFPs and their corresponding targets or modes of action are provided. The list of peptides and targets is non-exhaustive and serves to illustrate the mechanistic diversity by which AFPs exert their activity. Figure created in BioRender. Odabas, M. (2026) https://BioRender.com/h8i48hg.

The antifungal mechanisms of AFPs are diverse (Figure 2) and, in some cases, remain partially or entirely unclear. Still, some AFPs seem to specifically target fungi (*e.g.*, echinocandins, histatins) while others show a broader antimicrobial activity that also targets bacteria and viruses (*e.g.*, magainins, mammalian defensins). Of note, some AFPs seem to have multiple modes of action 27.

The cell wall is a characteristic feature of fungal cells and essential for structural integrity of the cell, protection against environmental stress and a regulatory portal for interactions with the extracellular room. It is mainly composed of glucans, chitin, mannans and glycoproteins 45. Thereby, chitin and β -glucans are the major structural components of many fungal cell walls, thus representing molecular targets of several fungi-specific AFPs. For instance, echinocandins are non-competitive inhibitors of (1–3)- β -D-glucan synthase, including clinically relevant representatives like caspofungin, micafungin, or anidulafungin 46. Another example is the killer toxin WmKT, from the yeast *Cyberlindnera mrakii*, which also inhibits β -1,3-glucan synthesis 47*.* Other AFPs target chitin synthesis, including the nikkomycins, of which nikkomycin Z, a secondary metabolite produced by *Streptomyces tendae*, is the most studied one 48, 49. Cell wall-resident mannans, polysaccharides primarily composed of D-mannose monomers, can be specifically bound by actinomycete-produced pradimicins 50 and benanomicins 51.

The distinct characteristics of the fungal membrane represent another intervention site. In fact, membrane disruption is a central strategy in antifungal therapy, with two major clinically relevant drug classes relying on this mechanism. Azoles inhibit the fungal enzyme lanosterol 14 α -demethylase, disrupting ergosterol synthesis and causing toxic sterol intermediate accumulation, while polyenes like amphotericin B bind to ergosterol in the fungal cell membrane, forming membrane pores that lead to ion leakage and cell death. Along these lines, some AFPs induce membrane damage through pore formation. For example, RsAFP2, isolated from radish seeds (*Raphanus sativus*), targets fungus-specific membrane glucosylceramides. Similarly, the insect defensin heliomycin also interacts with glucosylceramides, but through different motifs. Other plant defensins like DmAMP1 from dahlia or Psd1 from peas, also act on fungal-specific sphingolipids 52, while tobacco NaD1, tomato TPP3, barrel clover MtDef4 or coral bells HsAFP1 affect fungal phospholipids 53, 54. Upon lipid interaction, these defensins are either internalized or remain on the outside, but in either case, downstream effects are triggered that promote cytotoxicity. To which extent membrane permeabilization occurs as a non-specific secondary effect following specific intracellular targets remains to be more clearly elucidated.

As mentioned, some plant defensins are internalized after interaction with the cell membrane, and appear to subsequently interact directly with intracellular targets. For instance, upon entering the filamentous fungus *Neurospora crassa*, Psd1 localizes within the nucleus and acts on a nuclear cyclin-like protein involved in cell cycle regulation 55. Other examples that require peptide internalization for antifungal execution include NaD1 56 and MtDef4 57, although the exact molecular targets remain to be identified. The mammalian peptide Histatin 5, which is internalized through transporters (not endocyted), affects the mitochondrial membrane of *C. albicans* and disrupts respiratory metabolism, resulting in ATP release and cell death 58.

Intriguingly, several AFPs have been shown to engage the regulated cell death (RCD) machinery of fungal pathogens 59. Unicellular organisms do possess RCD mechanisms that are in part reminiscent of those in higher eukaryotes. They play a crucial role in maintaining homeostasis and fitness of the clonal population by helping to selectively eliminate damaged, dysfunctional or otherwise unneeded cells 60–62 under different physiological scenarios, including colony differentiation and aging, among others 63. Much of the research on fungal RCD, particularly in mechanistic terms, has focused on the baker’s yeast *Saccharomyces cerevisiae* 60, 64, 65. Pathogenic yeasts, such as *Candida* species, and molds like *Aspergillus* species, have both been shown to exhibit typical morphological markers of apoptosis in response to specific stressors 66, although other RCD subroutines, such as autophagic cell death and regulated necrosis, remain largely unexplored.

Most of the work on RCD in pathogenic fungi has been performed in *Candida* spp. For instance, classical apoptotic triggers like acetic acid and hydrogen peroxide 67 can induce RCD in *Candida* spp. Importantly, *Candida* spp. code for a metacaspase, which has been causally linked to different lethal scenarios 68, 69, including oxidative stress, exposure to the quorum-sensing molecule farnesol, amphotericin B 67, 70 and micafungin 71 treatment. Interestingly, host cells appear capable of harnessing the apoptotic mechanisms of fungal pathogens as a means of defense: for instance, murine macrophages have been shown to promote metacaspase activation and apoptosis in *C. albicans* 72. This observation exemplifies the potential of external RCD activation as an antifungal route.

Indeed, a number of AFPs have been demonstrated to elicit RCD in pathogenic fungi. For instance, the plant AFPs RsAFP2 73 and HsAFP1 74 induce morphological markers of apoptosis in *C. albicans*, including production of reactive oxygen species (ROS), phosphatidylserine externalization and apoptotic DNA fragmentation. While HsAFP1-mediated RCD seems to involve the induction of mitochondrial dysfunction 74, the lethal activity of RsAFP2 73 involves ceramide accumulation 75. Ceramides are linked to apoptotic pathways across various species, including *S. cerevisiae*, where they function independently of the only reported metacaspase, Yca1 61, 76. Interestingly, RsAFP2-induced RCD in *C. albicans* does also not rely on CaMca1, the sole metacaspase identified in *C. albicans* 75. However, RsAFP2 treatment results in measurable caspase activity, suggesting the involvement of additional (as yet to be identified) metacaspases or caspase-like proteases. Other AFPs have been shown to exhibit apoptotic markers concomitant with the activation of (meta)caspase activity in *C. albicans*, *e.g.*, diverse insect-derived peptides like a variant of coprisin 77 from the dung beetle, psacotheasin from longhorn beetle larvae 78 or periplanetasin-2 from the American cockroach 79. However, a causal link between metacaspase activation and cell death execution was not explicitly explored.

Lactoferrin (LF), a multifunctional iron-binding glycoprotein found abundantly in mammalian milk and various exocrine secretions, has also been connected to fungal RCD. Beyond its role in iron homeostasis, it exhibits broad-spectrum antimicrobial properties, including notable antifungal activities 80 against various pathogenic species like *Candida* spp. and *Cryptococcus neoformans*. Cell death induced by LF exhibits morphological markers of apoptosis 81 and is mediated by inhibition of the plasma membrane H ${}^{+}$ -ATPase Pma1, the major regulator of cytosolic pH in fungi, resulting in subsequent disturbance of ion homeostasis and mitochondrial dysfunction 82. Whether metacaspase activity is required for the lethal effects remains unknown, but at least in *S. cerevisiae*, human LF-triggered apoptosis is dependent on functional mitochondria and Yca1 83.

## SYNERGISTIC POTENTIAL OF AFPs IN COMBINATION WITH CONVENTIONAL ANTIFUNGAL AGENTS

In addition to their standalone antifungal potential, AFPs may also serve as potentiators of conventional antifungal agents to achieve synergistic effects. This kind of combinatorial approach allows for reduced doses of the combined agents while achieving an antifungal outcome comparable (additive) or superior (synergistic) to single-compound treatment. This may allow tackling recurrent or chronic fungal infections, where existing therapies prove inadequate at higher doses due to host toxicity constraints or side effects. Additionally, it may be effective against strains resistant to current treatment dosages and help minimize the likelihood of resistance development overall.

The mechanistic basis for such combinatorial effects may rely on one or more complementary activities achieved by the combination. These may include, for example, enhanced permeability. As mentioned above, many AFPs are able to compromise the integrity of fungal cell walls and/or cell membranes, which may allow for a more effective entry of the second antifungal drug. AFPs may also amplify stress responses by inducing cellular stress or oxidative damage (see above), weakening the pathogen and allowing the second compound to capitalize on this vulnerability. Other mechanisms may include the inhibition of compensatory, adaptive or resistance pathways by one compound that have been activated by the pathogen to counteract the effects of the other compound.

Numerous studies indicate that AFPs hold great potential as candidates for such combinatorial approaches 84, 85. For instance, LF-derived peptides potentiate fluconazole activity against various *Candida* species, including azole-resistant mutants 86, 87. While the synergistic mechanism remains unclear, it does not seem to involve an LF-mediated increase in intracellular azole uptake 88. This effect is also present in combination with other azole drugs 89 as well as, to a more limited extent, with amphotericin B 89 and 5-fluorocytosine. In *Cryptococcus* spp., LF also seems to exhibit synergistic effects, especially with amphotericin B 90. Of note, the LF derivative hLF1-11 was recently shown to exert combinatorial activity with fluconazole and the echinocandin anidulafungin in *Candida* and NAC strains 91.

Another example is LL-37, an AFP derived from the human cathelicidin hCAP18 that exhibits significant antifungal activity against various pathogenic fungi, including *C. albicans* and *Aspergillus* species 92. A recent study showed evidence for strong synergistic effects of LL-37 in combination with either fluconazole, amphotericin B or caspofungin in various clinical isolates of *C. auris* 93. Furthermore, human β -defensin-3 has demonstrated potent fungicidal activity against *C. auris*, and when combined with caspofungin, exhibited complete synergy across various clinical isolates. This synergistic interaction enhances membrane permeability, leading to increased efficacy of the antifungal treatment 94.

A number of truncated versions of specific peptides have also been shown to bear combinatorial activity. For instance, a human salivary mucin 7-derivative (MUC7 12-mer) in combination with miconazole showed *in vitro* synergism against *C. neoformans* and additive effects against *C. albicans* 95. Moreover, specific fragments of crotalicidin (Ctn 15–34), present in the venom of a South American rattlesnake, synergized *in vitro* with AmpB 96, and the N-terminus truncated isoform of human hepcidin 25 (Hep-20) 97 showed synergistic effects with several antifungals against *N. glabrata*. Further examples of AFPs with combinatorial potential include ranalexin from bullfrog (*Rana catesbeiana*) skin, *Dermaseptin S3 (DS3)* from the South American frog *Phyllomedusa sauvagii*, or magainin 3 98, for which strong *in vitro* synergistic effects with the echinocandins caspofungin or anidulafungin were observed against both *C. albicans* and *N. glabrata*. However, upon testing one of the identified combinations (caspofungin-ranalexin) in a murine model of disseminated candidiasis, no synergistic inhibition of *C. albicans* was observed 98. In addition, ranalexin treatment might have compromised animal welfare 98. These results exemplify the necessity of *in vivo* validation despite promising *in vitro* activities.

## IMMUNOMODULATORY ACTIVITY

While any individual may potentially suffer from fungal infections, people with weakened immune systems are at highest risk of severe illness and death. This group includes, for example, HIV/AIDS patients, those undergoing cancer chemotherapy, and individuals on immunosuppressive medications, for example, following organ transplants. In addition, the age-related dysfunction of the innate and adaptive immune system (immunosenescence) increases infection susceptibility in the elderly 99, a population group that is steadily increasing globally due to the ongoing rise in life expectancy over recent decades. In turn, this underscores the critical role of a fully functioning host immune response in managing mycoses. This observation also points to immunomodulation as a promising strategy for developing new treatments for disseminated fungal diseases 100. In line with this, recent research has explored the use of immunomodulating agents as adjunctive therapies to conventional antifungal drugs 101. This dual approach aims to enhance the patient’s immune response, particularly in those with weakened immunity, to more effectively combat and eradicate fungal infections.

In that sense, it is interesting to explore whether some AFPs might intrinsically bare immunomodulatory capacities themselves. In fact, several of the few AFPs having reached the clinical trial stage have also been attributed such activities, including hLF1-11 or the synthetic peptide CZEN-002 102.

Another well-documented example is LL-37, the precursor of which (hCAP18) is primarily produced by epithelial cells and neutrophils 103. Besides its direct antifungal activity (see above), it has also been shown to play important immunomodulatory roles. For instance, LL-37 promotes the release of pro-inflammatory cytokines and chemokines 104, enhances neutrophil recruitment 105, and modulates macrophage activity 106, contributing to a more robust immune response against fungal pathogens. In addition, α - 107, 108 and β -defensins 109 directly influence cytokine production and the adaptive immune system. Notably, distinct β -defensins have also been shown to chemoattract CD4 T cells and immature dendritic cells 110 as well as monocytes 111 and macrophages 112, 113. While these observations suggest that human defensins bridge the innate and adaptive immune system, their specific effects are complex and situated at the intersection of numerous molecular pathways, thus requiring further investigation. This complexity is well reflected, for example, by the number of genes differentially expressed (1779) in macrophages stimulated with the TLR4 agonist KDO2-lipid A upon exposure to the β -defensin hBD3 and the connected up-/downregulated pathways 114. Furthermore, different histatins have been connected to cell-migratory activities, especially in the context of wound healing 115, 116, and further inflammatory responses. Histatin 3, for example, shows inhibitory activities on Toll-like receptor signaling and the activation of inflammatory cytokine production, at least in gingival fibroblasts 117. On the other hand, Histatin 1 stimulates chemokine and inflammatory cytokine secretion in fibroblasts and keratinocytes of the skin and gums 118. Histatin 1 was further shown to reduce lipopolysaccharide-induced nitric oxide production, inflammatory cytokine production, and inflammatory signaling in macrophages 119.

This immunomodulatory potential is not restricted to human AFPs. Although currently rather restricted, mounting data point towards similar effects for some plant defensins 101. For instance, Lc-def, an AFP isolated from the seeds of the lentil *Lens culinaris* that inhibits growth of different *Candida* spp. 120, can impact cytokine response in different human immune cells 121. Another example is Psd1, which affects cytokine response in an epithelial–immune cell co-culture model upon *C. albicans* infection 122. Similarly, NaD1 impacts cytokine and chemokine production in various immune cells 123. Of note, the latter studies also employed Caco-2 cells, which are derived from intestinal epithelial cells, to demonstrate that Psd1 and NaD1 possess the ability to penetrate epithelial barriers. Transepithelial absorption in the human gut is crucial for enabling direct immune cell modulation or epithelial-mediated signaling and thus represents an additional asset upon assessing (plant) AFP activities. While *in vivo* data is certainly needed to support these observations, they highlight the opportunities in exploring the immunomodulatory potential of plant-derived AFPs that also exhibit antifungal properties.

Thus, a number of examples showcase that at least some human as well as non-human AFPs possess a unique dual activity, acting both as direct antifungals and immunomodulatory agents. Although at an exploratory stage, this may represent a powerful combination to allow for simultaneous targeting from two fronts, pharmacological pathogen clearance and increased immune engagement.

## CONCLUSION

In conclusion, AFPs represent a promising class of therapeutic agents with significant potential for combating fungal infections, especially in light of continuously rising antifungal resistance. The mechanistic routes by which different AFPs exert their antifungal properties are beginning to be understood with much more remaining to be learned in this respect. As our understanding of these processes expands, so too will (i) the therapeutic potential of AFPs, particularly in terms of targeted treatment and efficacy against specific fungal infections, (ii) minimization of side effects by designing treatment strategies that reduce adverse interactions, (iii) mechanisms of interaction with other drugs in combination therapies, (iv) prediction of resistance mechanisms to circumvent or delay the onset of resistance, also accounting for host genetics and the host microbiome (personalized therapy), and (v) optimization of drug design and development in the frame of medicinal chemistry efforts to improve the compound’s affinity, selectivity, and efficacy. In fact, while this short piece centered mostly on natural AFPs, synthetic and semisynthetic AFP variants also demonstrate considerable promise 84. This is not only true regarding the standalone activity, but also their combinatorial potential 124. There is substantial potential in utilizing *in silico* peptide optimization to enhance naturally occurring peptides or design novel ones. Additionally, production can be optimized through recombinant techniques or chemical synthesis 84. Advancements in computational methods, including predictions on drug target specificity and resistance development, will further enhance this potential, offering relatively cost-effective strategies to advance the field. In particular, the integration of machine learning and artificial intelligence (AI) holds considerable promise and may, for example, propel the *de novo* design of AFPs 125–128. Importantly, aspects like the systematic assessment of physicochemical properties, drug target identification, and the prediction of drug–target interactions, will enable the rational optimization of both novel and established AFPs. Furthermore, artificial intelligence-driven approaches could support personalized drug development by tailoring therapeutic design to specific patient cohorts, depending on the availability of relevant clinical and molecular data. This potential applies both to the standalone activity of AFPs as well as to their capacity to synergistically enhance both the activity of known antifungals and the patient’s immune response. Thus, AFPs enrich our antifungal armamentarium with a multi-pronged tool that may lead to more effective and versatile treatments.

## CONFLICT OF INTEREST

The authors declare no conflict of interest.

## ABBREVIATIONS

AFP – antifungal peptide

FIs – fungal infections

LF – lactoferrin

RCD – regulated cell death

NAC – non-albicans *Candida*
